# How to trigger a fungal weapon

**DOI:** 10.7554/eLife.10504

**Published:** 2015-09-01

**Authors:** Hubertus Haas

**Affiliations:** Division of Molecular Biology, Biocenter, Medical University of Innsbruck, Innsbruck, Austriahubertus.haas@i-med.ac.at

**Keywords:** *Aspergillus terreus*, terrein, methionine, nitrogen starvation, iron limitation, siderophores, other

## Abstract

A fungus called *Aspergillus terreus* produces a secondary metabolite in response to various environmental signals to give it an advantage over its competitors.

**Related research article** Gressler M, Meyer F, Heine D, Hortschansky P, Hertweck C, Brock M. 2015. Phytotoxin production in *Aspergillus terreus* is regulated by independent environmental signals. *eLife* **4**:e07861. doi: 10.7554/eLife.07861

**Image** Researchers have identified several genes that trigger the production of terrein (pink) in the fungus

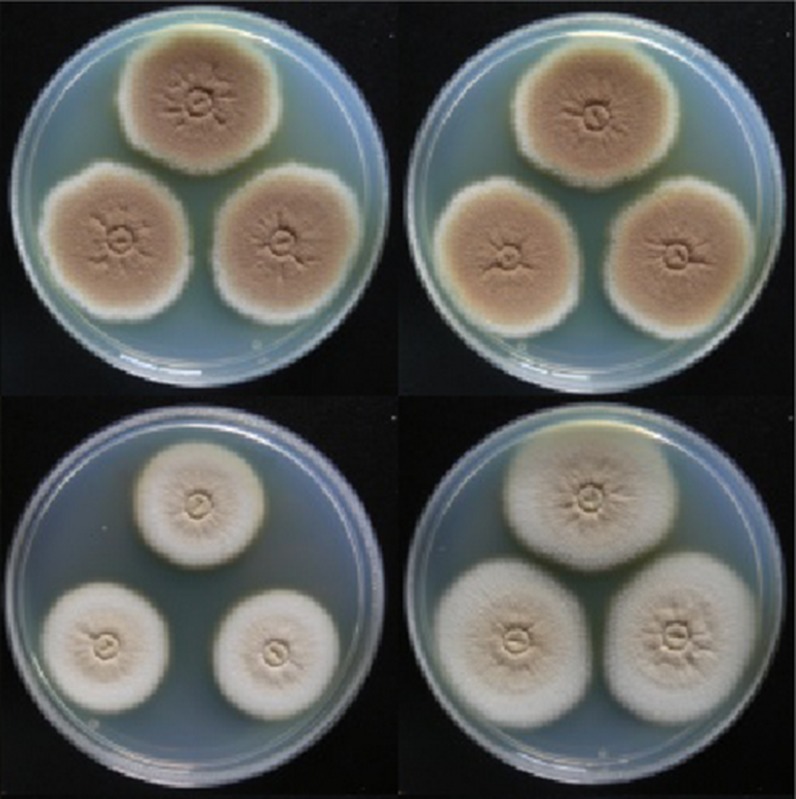

Fungi affect our lives in many different ways, both positive and negative. One of the reasons for this is that most fungi produce a multitude of small organic molecules called secondary metabolites. Different species employ a strikingly diverse arsenal of secondary metabolites, most of which are released into the environment (Sanchez et al., 2012). Secondary metabolites are not directly required to ensure the growth of the organism, but confer an advantage under specific environmental conditions.

Fungi use secondary metabolites to defend against predators and competitors, for chemical communication, or in the case of pathogenic fungi, to manipulate their animal and plant hosts (Brakhage et al., 2013). Secondary metabolism is therefore likely to be shaped to a large extent by interactions with other organisms. For example, fungi secrete enzymes to digest their food, which allows them to grow on virtually any organic matter, but also means that the products of their digestion are in principle a free meal for other organisms. And by secreting secondary metabolites that target these organisms, fungi are able to defend their niche to avoid competitors taking advantage of the available food.

Well-known examples of secondary metabolites produced by fungi are the poisonous food contaminant aflatoxin, the antibiotic penicillin and the anticancer drug taxol. These molecules illustrate the negative and positive effects of secondary metabolites on humans, and underline their outstanding potential for medicinal use. However, it is not known what roles most of these molecules play in the lives of the fungi that produce them. Moreover, most secondary metabolites are not produced when the fungi are grown in the laboratory, which makes it difficult to characterize them. Now in *eLife*, Matthias Brock and co-workers – including Markus Gressler as first author – report a new role for a major secondary metabolite called terrein, and characterize the environmental stimuli that induce the mold *Aspergillus terreus* to produce it (Figure 1; Gressler et al., 2015).

**Figure 1.**
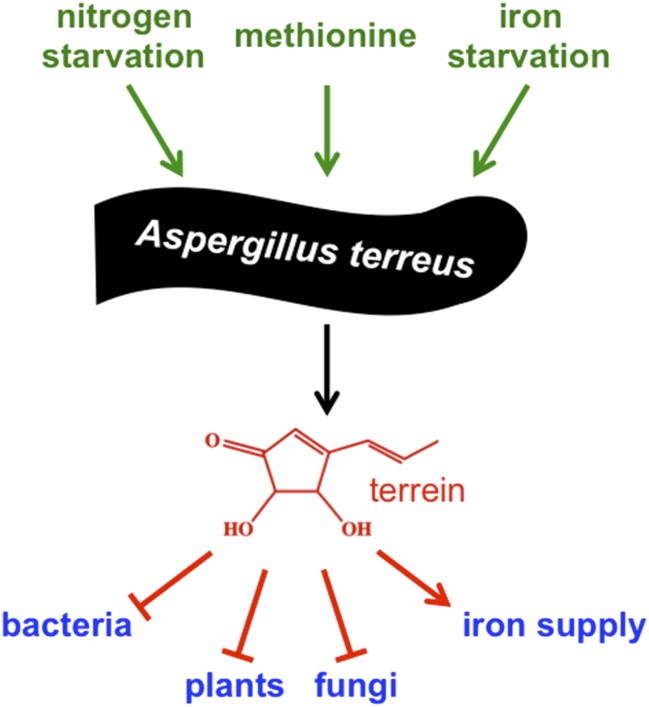
Environmental signals activate production of terrein by the mold *Aspergillus terreus* to improve its competitiveness. To adapt to changing environmental conditions and different ecological niches, microorganisms need to be able to sense and respond to environmental signals. Gressler et al. identified three independent signals that stimulate production of the compound terrein by *Aspergillus terreus* – nitrogen starvation, methionine, and iron starvation. In this mold's natural niche within plants and in the soil surrounding plant roots, terrein is a chemical weapon used to inhibit the growth of bacteria, plants and other fungi, but also helps to improve iron supply to the producer.

*A. terreus* is a common soil-borne fungus that feeds on dead organic material, but is also able to invade plants and cause life-threatening infections in humans with weakened immune systems. Genome analysis indicated that this fungus might produce more than 68 secondary metabolites, although only 14—including the cholesterol-lowering drug lovastatin—have been identified so far (Guo and Wang, 2014). The compound terrein was first described 80 years ago, but how *A. terreus* makes terrein was only resolved in 2014 by the Brock group (Zaehle et al., 2014). Terrein was previously shown to be harmful to plant cells as it inhibits the germination of seeds and causes lesions on plant surfaces, and probably helps the fungus to colonize its host.

Based on the observation that potato extract (an ingredient of a standard medium used for culturing fungi) activates the production of terrein, Gressler et al. – who are based at the Hans Knoell Institute, Friedrich Schiller University and Nottingham University – systematically characterized how different conditions impact terrein production. This analysis revealed that the genes that encode the terrein biosynthetic pathway are activated by three independent environmental stimuli: nitrogen starvation, iron starvation, and the presence of the amino acid methionine. These conditions are typically found in the plant and the plant root area, known as the rhizosphere, and are used by the mold to sense these niches.

Next, by genetic engineering of the mold, Gressler et al. identified three transcription factors that activate genes in response to environmental signals. Previous studies have revealed the roles of these regulators in altering the production of primary metabolites – which are required for normal growth and reproduction – in response to stress and the availability of nitrogen and iron (Haas, 2012; Tudzynski, 2014). However, it is not known how the mold perceives the methionine signal. Nitrogen and iron also regulate the production of other secondary metabolites (Tudzynski, 2014; Wiemann et al., 2014), suggesting that these environmental cues are often used to adjust secondary metabolism. The complex environmental control of terrein production revealed by Gressler et al. represents a prime example of how microorganisms adapt their secondary metabolism to the niche they inhabit.

In addition to its ability to inhibit the growth of plants, it has been reported that terrein can inhibit the growth of bacteria, fungi and mammalian cells, and that it can also act as an antioxidant and anti-inflammatory (Zaehle et al., 2014). Now, Gressler et al. have discovered that terrein supports iron uptake by the fungus that produces it, but inhibits the growth of even closely related molds. This clearly indicates that terrein improves the competiveness of the producer. It will be exciting to learn how terrein is able to influence many different biological processes in different organisms, and how the producer protects itself against this molecule.
